# Peste Des Petits Ruminants Virus Infection Induces Syncytium Formation via RhoA-Rock1 Signaling Pathway to Promote Viral Replication

**DOI:** 10.3390/ani16152405

**Published:** 2026-08-04

**Authors:** Wei Li, Hongnuan Wang, Chenyu Song, Rong Pang, Hanwei Yin, Mengyuan Liu, Xiaozhu Yang, Zilong Sun, Ding Zhang, Jingyu Wang, Bo Wen, Bo Yang

**Affiliations:** 1 Shanxi Key Laboratory of Animal Disease Research, Prevention and Control, College of Veterinary Medicine, Shanxi Agricultural University, Taigu, Jinzhong 030801, China; 2 College of Veterinary Medicine, Shanxi Agricultural University, Jinzhong 030801, China; 3 College of Veterinary Medicine, Northwest A&F University, Yangling 712100, China; 4 College of Animal Science and Veterinary Medicine, Henan Institute of Science and Technology, Xinxiang 453000, China

**Keywords:** peste des petits ruminants virus, syncytium formation, RhoA-Rock1 signaling pathway, viral replication

## Abstract

Peste des petits ruminants (PPR) is an acute, severe, and highly contagious disease caused by peste des petits ruminants virus (PPRV), which primarily induces tissue damage through the formation of syncytia. Therefore, elucidating the molecular mechanism underlying PPRV-induced syncytium formation is of critical importance. In this study, we generated Vero CCL-81 cell lines stably expressing the PPRV receptors SLAM (lymphocytic) or Nectin-4 (epithelial) and further found that PPRV infection significantly induced syncytium formation in these cell lines as well as in goat mammary epithelial cells (GMECs) and ovine rumen epithelial cells (ORECs). Furthermore, the viral H and F proteins served as the pivotal determinants of syncytium formation. More importantly, we discovered that PPRV-induced syncytium formation is dependent on the RhoA-Rock1 signaling pathway and that inhibition of syncytium formation markedly suppressed PPRV replication. Collectively, our findings provide novel insights into the molecular mechanism of PPRV-induced syncytium formation, offer theoretical references for the development of new antiviral strategies, and advance the scientific eradication of PPR.

## Simple Summary

Syncytium formation represents a critical mechanism through which peste des petits ruminants virus (PPRV) infection induces tissue damage. Thus, elucidating the molecular basis of this process is essential for controlling PPRV infection. In this study, we found that PPRV infection significantly induced syncytium formation in various cell types and that the viral H and F proteins served as the key determinants. Furthermore, we demonstrated that PPRV-induced syncytium formation was dependent on the Ras homolog family member A (RhoA)/Rho-associated coiled-coil-containing protein kinase (ROCK1) signaling pathway and that inhibition of syncytium formation markedly suppressed viral replication. Collectively, our findings confirm the mechanistic role of RhoA/ROCK1 in PPRV-induced syncytium formation and establish a theoretical basis for the development of new antiviral strategies.

## 1. Introduction

Peste des petits ruminants (PPR) is an acute, highly contagious disease caused by the peste des petits ruminants virus (PPRV). It primarily affects goats, sheep, and wild ruminants such as deer and antelopes, with typical clinical manifestations including acute pneumonia, diarrhea, and oral erosion [1]. The disease is characterized by high morbidity, a short disease course, and a mortality rate exceeding 90%. The World Organisation for Animal Health (WOAH) has listed PPR as a notifiable animal disease and aims to achieve global eradication of PPR by 2030. Currently, the control of PPR predominantly relies on immunization with live attenuated vaccines; however, the poor stability of these vaccines may lead to insufficient herd immunity, thereby increasing the risk of disease outbreaks. Therefore, further in-depth studies on the pathogenic mechanisms of PPRV are urgently needed to support the development of more stable and effective vaccines.

Syncytium formation is a cytopathic effect (CPE) characterized by the fusion of infected cells with neighboring cells, leading to the formation of multinucleated giant cells. This process is primarily mediated by viral fusion proteins expressed on the surface of virions, which trigger membrane fusion upon interaction with host receptors [2]. Syncytium formation is commonly observed in infections caused by various enveloped viruses, including those of the families Paramyxoviridae, Retroviridae, Herpesviridae, and Coronaviridae [3,4,5]. The membrane fusion activity of viruses typically requires the cleavage and activation of viral glycoproteins by host proteases and is regulated by multiple fusion-related signaling pathways [6,7]. Syncytium formation facilitates direct cell-to-cell viral spread, thereby allowing the virus to evade extracellular neutralizing antibodies and host immune surveillance [8]. Moreover, syncytia contribute to tissue damage, inflammatory responses, and increased disease severity [4]. Therefore, an in-depth understanding of the molecular mechanisms underlying syncytium formation holds promise for identifying new targets for antiviral interventions and vaccine development. As an important member of the genus *Morbillivirus*, PPRV induces typical syncytium formation upon infection of host cells. Our previous studies have preliminarily demonstrated that pharmacological inhibition of syncytium formation significantly suppresses PPRV replication [9], indicating that the induction of syncytium formation is a key mechanism by which PPRV promotes its own proliferation. However, how PPRV infection triggers syncytium formation remains unclear.

Similar to other viruses, PPRV establishes infection through the binding of its surface glycoproteins to receptors on host cells. To date, SLAM (signaling lymphocyte activation molecule) and Nectin-4 have been identified as the primary receptors for PPRV [10]. SLAM is predominantly expressed on lymphocytes and serves as the core receptor for PPRV infection in these cells, whereas Nectin-4 is mainly expressed on epithelial cells and acts as the entry receptor for PPRV infection of epithelial cells. Here, we established Vero CCL-81 cell lines stably expressing goat SLAM and Nectin-4, respectively, via lentiviral packaging technology. These cell lines not only support efficient PPRV replication in vitro but also enable the direct observation of syncytium formation, making them an ideal model for studying the mechanisms underlying PPRV-induced syncytium formation.

RhoA is an important member of the Rho GTPase family and plays a central role in regulating cytoskeletal rearrangement, actin contraction, cell migration, and vesicular transport by acting on its downstream effector molecule Rock1. Recent studies have shown that the RhoA-Rock1 signaling pathway is extensively involved in various viral infection processes, particularly playing critical roles in virus-induced syncytium formation and viral entry. Regarding syncytium formation, Rock1 enhances actin contractility and membrane tension by phosphorylating myosin light chain (MLC), thereby promoting membrane fusion between infected and neighboring cells. It has been reported that RhoA interacts with the fusion glycoprotein of respiratory syncytial virus (RSV) to facilitate virus-induced syncytium formation [11,12]. The P10 protein of avian reovirus (ARV) promotes MLC expression and phosphorylation by activating RhoA and Rac1, thereby inducing syncytium formation and promoting viral replication [13]. Regarding viral entry, it has been reported that infectious bursal disease virus (IBDV) induces actin rearrangement by activating the RhoA signaling pathway to facilitate viral entry [14]. Newcastle disease virus (NDV) activates the RhoA signaling pathway through an Src-dependent mechanism, which in turn activates downstream MLC to induce actin rearrangement and promote viral entry [15]. Taken together, the RhoA-Rock1 signaling pathway is one of the core regulatory pathways involved in virus-induced syncytium formation. However, whether it plays a key regulatory role in syncytium formation induced by PPRV infection remains to be further investigated.

In this study, we elucidate the important role of the RhoA-Rock1 signaling pathway in syncytium formation induced by PPRV infection and its impact on PPRV replication. The results will unravel the molecular mechanism underlying syncytium formation induced by PPRV infection and provide a new theoretical basis for the prevention and control of PPRV infection.

## 2. Materials and Methods

### 2.1. Cell and Virus Preparation

Vero CCL-81 cells were prepared in our lab and cultured in Dulbecco’s modified Eagle medium (DMEM) with high glucose (Gibco, New York, NY, USA). GMECs and ORECs were purchased from Shanghai Anwei Biotechnology Co., Ltd. (Shanghai, China). The cells exhibited typical epithelial-like morphology and were cultured in Dulbecco’s Modified Eagle Medium/Nutrient Mixture F-12 Ham (DMEM/F-12) (Gibco, New York, USA). At 90% confluence, the cells were subcultured at a ratio of 1:2. The media were supplemented with 10% fetal bovine serum (Sunview, Shenzhen, China). Cells were maintained at 37 °C in a 5% CO_2_ incubator. The attenuated PPRV strain Nigeria 75/1 (GenBank accession no. HQ197753) was obtained from our laboratory culture collection. All cell infection experiments were performed at a multiplicity of infection (MOI) of 2.

The construction details for Vero CCL-81-SLAM and Vero CCL-81-Nectin-4 are as follows: HEK-293T cells at 60–70% confluence were transfected with pMD2.G, pSPAX.2, and the plasmid of interest at a ratio of 3:6:8. Lentivirus-containing supernatants were harvested at 48 h and 72 h post-transfection, pooled, and centrifuged at 4000 rpm for 7 min. The clarified supernatants were then adjusted to a final volume of 10 mL with DMEM supplemented with 10% fetal bovine serum, and Polybrene was added at a dilution of 1:1000. Vero CCL-81 cells were subsequently infected with the viral supernatants in two rounds. After infection, the cells were trypsinized, passaged, and subjected to continuous selection with puromycin (4 μg/mL) for three passages to establish stable cell lines.

### 2.2. Antibodies

Primary antibodies used in this study were as follows: mouse anti-GAPDH (Proteintech, Wuhan, China, 60004-1-Ig), rabbit anti-Flag (Proteintech, 20543-1-AP), rabbit anti-Rock1 (Proteintech, 66782-1-Ig), rabbit anti-RhoA (Affinity, Nanjing, China, AF6352), mouse anti-PPRV-N monoclonal antibody (provided by the China Animal Health and Epidemiology Center, Qingdao, China), and mouse anti-PPRV-H and anti-PPRV-V antibodies (prepared in our lab).

### 2.3. Western Blot Assay

Cells were lysed in radioimmunoprecipitation assay (RIPA) lysis buffer supplemented with protease inhibitors. After quantification, equal amounts of protein were separated by sodium dodecyl sulfate–polyacrylamide gel electrophoresis (SDS-PAGE) and transferred onto PVDF membranes. Membranes were blocked with 5-10% non-fat milk in Tris-buffered saline with Tween 20 (TBST) for 2 h, then incubated with primary antibodies against PPRV-N, PPRV-V, RhoA, Rock1, Flag, and GAPDH overnight at 4 °C. After washing, membranes were incubated with horseradish peroxidase (HRP)-conjugated secondary antibodies for 1 h. Signals were detected using an enhanced chemiluminescence (ECL) system and quantified using ImageJ software (version 1.54d), with GAPDH as the internal control.

### 2.4. Immunofluorescence Assay

PPRV-infected or plasmid-transfected cells were fixed with 4% paraformaldehyde (Solarbio, Beijing, China) for 30 min, permeabilized with 0.1% Triton X-100 (Sigma-Aldrich, St. Louis, MO, USA) for 10 min, and blocked with 10% non-fat milk for 1 h. Primary antibodies against PPRV-H or Flag were applied overnight at 4 °C. After washing, cells were incubated with Alexa Fluor 488 AffiniPure Goat Anti-Rabbit IgG (H + L) (FUSHENBIO, Shanghai, China) for 1 h and stained with 4′,6-diamidino-2-phenylindole (DAPI) (Solarbio, China) for 5 min. Fluorescent images were captured using a fluorescence microscope and analyzed using ImageJ software.

### 2.5. RNA Interference

siRNAs targeting RhoA (siRhoA-1: 5′-GAAGUCAAGCAUUUCUGUC-3′; siRhoA-2: 5′-AAGGCAGAGAUAUGGCAAA-3′) and a scrambled control siRNA were synthesized by GenePharma (Beijing, China). Logarithmic-phase Vero CCL-81-gSLAM cells were seeded in 24-well plates. At 60% confluence, cells were transfected with siRNAs (50 nM final concentration) using JetPRIME (Polyplus, Illkirch-Graffenstaden, France) according to the manufacturer’s instructions. At 24 h post-transfection, cells were infected with PPRV (MOI = 2) for 24 h. Cell lysates and culture supernatants were collected for analysis. RhoA knockdown efficiency was confirmed by Western blotting.

### 2.6. Syncytium Observation and Quantification

Cell morphology and syncytium formation were observed using a light microscope (Leica, Wetzlar, Germany) at the indicated time points. Syncytia were defined as multinucleated fused cells. The fusion index was calculated as the total number of nuclei within syncytia divided by the total number of syncytia. Syncytium number and the number of nuclei per syncytium were counted visually. The relative area of syncytia was measured with ImageJ software.

### 2.7. Plasmid Co-Transfection Assay

For single transfection groups, cells were transfected with 2 μg of F-Flag or H-Flag plasmid individually. For the co-transfection group, cells were co-transfected with 1.5 μg of F-Flag plasmid and 1.5 μg of H-Flag plasmid. Transfection was carried out using Lipofectamine 3000 according to the manufacturer’s protocol. At 48 h post-transfection, syncytium formation was observed under a microscope, and the cells were fixed for nuclear staining. Meanwhile, a PPRV infection group was set up as a parallel control.

### 2.8. Fusion Inhibitory Peptide (FIP) Syncytium Inhibitor Experiment

Cells at 60% confluence were infected with PPRV for 2 h, either with or without FIP (10 μg/mL, dissolved in DMSO, sequence: Z-D-Phe-Phe-Gly-OH) (Nanjing Leon Biotechnology Co., Ltd., Nanjing, China). A mock-infected group was also included. After infection, all supernatants were removed and replaced with DMEM maintenance medium supplemented with 2% serum. At the indicated time points, both cells and supernatants were collected for subsequent assays.

### 2.9. Rock1 Inhibitor Experiment (Y-27632)

Cells were pretreated with 50 μM Y-27632 before PPRV infection. After 2 h post-infection, maintenance media containing 2% serum were replaced. Cells were harvested at the indicated time points for Western blot analysis and syncytium observation.

### 2.10. TCID_50_ Assay

Viral titers were determined by the TCID_50_ assay. Virus-containing supernatants were serially diluted 10-fold in DMEM containing 2% fetal bovine serum (FBS) (10^−1^ to 10^−10^). Confluent monolayers of host cells in 96-well plates were washed with phosphate-buffered saline (PBS), and 100 μL of each viral dilution was added to eight replicate wells. Cytopathic effect (CPE) was observed daily for 3–5 days. Viral titers were calculated using the Reed–Muench method.

### 2.11. Statistical Analysis

All data represent the mean ± standard deviation (SD) from three independent experiments. Statistical analysis was performed using GraphPad Prism v9.0. Differences between two groups were assessed by unpaired two-tailed Student’s *t*-tests, and one-way ANOVA was used for comparisons among three or more groups. Statistical significance is indicated as follows: *** *p* < 0.001; ** *p* < 0.01; * *p* < 0.05; not significant (ns), *p* > 0.05.

## 3. Results

### 3.1. PPRV Infection Induces Syncytium Formation in Multiple Cell Lines

**Figure 1:**
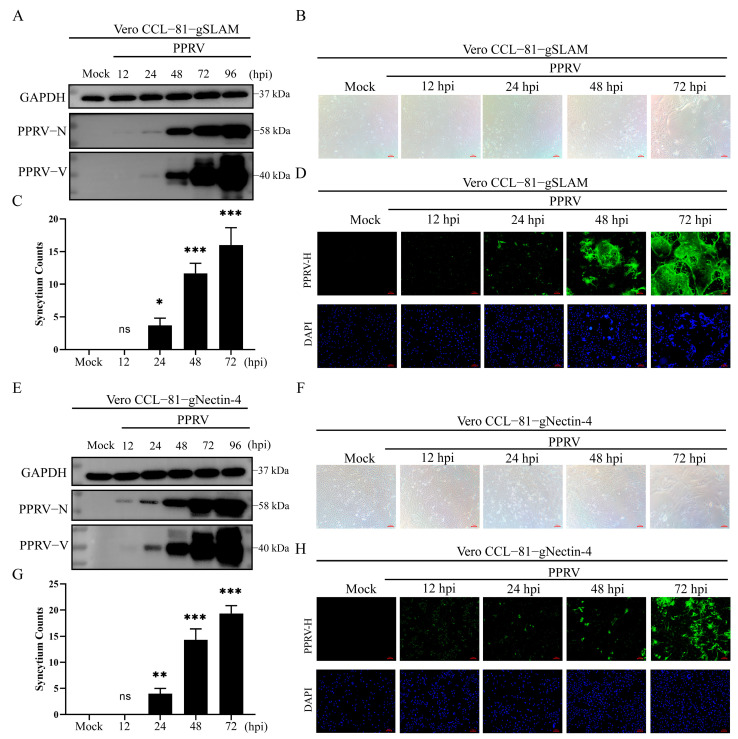
PPRV infection induces syncytium formation in Vero cell lines: (**A**,**E**) Western blotting analysis of PPRV-N and -V protein expression in Vero CCL-81-gSLAM (**A**) and Vero CCL-81-gNectin-4 (**E**) cells at different time points post-infection. (**B**,**C**) Representative bright-field microscopic images (**B**) and quantification (**C**) of syncytium formation in PPRV-infected Vero CCL-81-gSLAM cells. (**F**,**G**) Representative bright-field microscopic images (**F**) and quantification (**G**) of syncytium formation in PPRV-infected Vero CCL-81-gNectin-4 cells. (**D**,**H**) Immunofluorescence staining demonstrating syncytium formation in PPRV-infected Vero CCL-81-gSLAM (**D**) and Vero CCL-81-gNectin-4 (**H**) cells at the indicated time points. Green indicates PPRV H protein, and blue (DAPI) indicates nuclei. Scale bars: 100 μm. Data are representative of three independent experiments and presented as mean ± SD. ***, *p* < 0.001; **, *p* < 0.01; *, *p* < 0.05; ns, *p* > 0.05.

**Figure 2:**
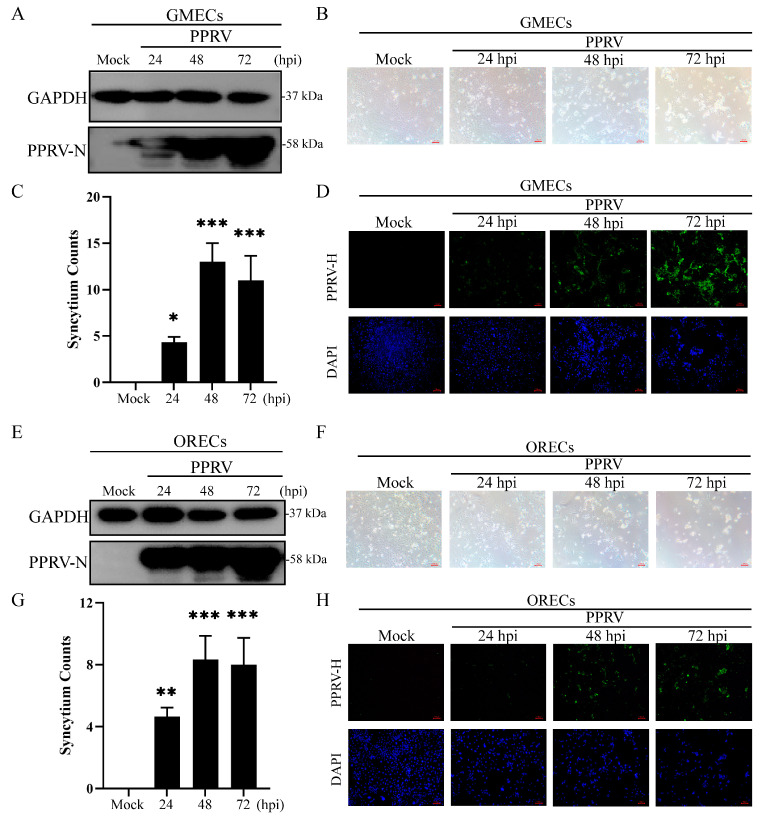
PPRV infection induces syncytium formation in primary host cells: (**A**,**E**) Western blotting analysis of PPRV-N protein expression in GMECs (**A**) and ORECs (**E**) cells at different time points post-infection. (**B**,**C**) Representative bright-field microscopic images (**B**) and quantification (**C**) of syncytium formation in PPRV-infected GMECs. (**F**,**G**) Representative bright-field microscopic images (**F**) and quantification (**G**) of syncytium formation in PPRV-infected ORECs. (**D**,**H**) Immunofluorescence staining demonstrating syncytium formation in PPRV-infected GMECs (**D**) and ORECs (**H**) cells at the indicated time points. Green indicates PPRV H protein, and blue (DAPI) indicates nuclei. Scale bars: 100 μm. Data are representative of three independent experiments and presented as mean ± SD. ***, *p* < 0.001; **, *p* < 0.01; *, *p* < 0.05.

To clearly observe syncytium formation upon PPRV infection, we established Vero CCL-81-gSLAM and Vero CCL-81-gNectin-4 cell lines stably expressing goat SLAM or Nectin-4 receptors, respectively. Western blotting and indirect immunofluorescence assays revealed that viral N and V protein expression was significantly upregulated following PPRV infection, indicating efficient viral proliferation in Vero CCL-81-gSLAM (Figure 1A) and Vero CCL-81-gNectin-4 (Figure 1E). Concurrently, the number of syncytia increased markedly in Vero CCL-81-gSLAM (Figure 1B,C) and Vero CCL-81-gNectin-4 (Figure 1F,G) cells. To further confirm the formation of syncytia, we performed immunofluorescence staining on the nuclei. The results showed that the blue fluorescence of cell nuclei gradually aggregated and fused into larger structures (Figure 1D,H), fully demonstrating that PPRV infection induces syncytium formation in these model cells. To further confirm this phenotype, we selected two additional cell types derived from the natural hosts of PPRV, GMECs and ORECs, as infection models. Consistent with the above results, PPRV proliferated efficiently in these cells (Figure 2A,E), and the number of syncytia significantly increased in a time-dependent manner following PPRV infection in GMECs (Figure 2B–D) and ORECs (Figure 2F–H). These findings collectively indicate that PPRV infection markedly induces syncytium formation.

### 3.2. PPRV H and F Proteins Are Key Viral Proteins Inducing Syncytium Formation

**Figure 3:**
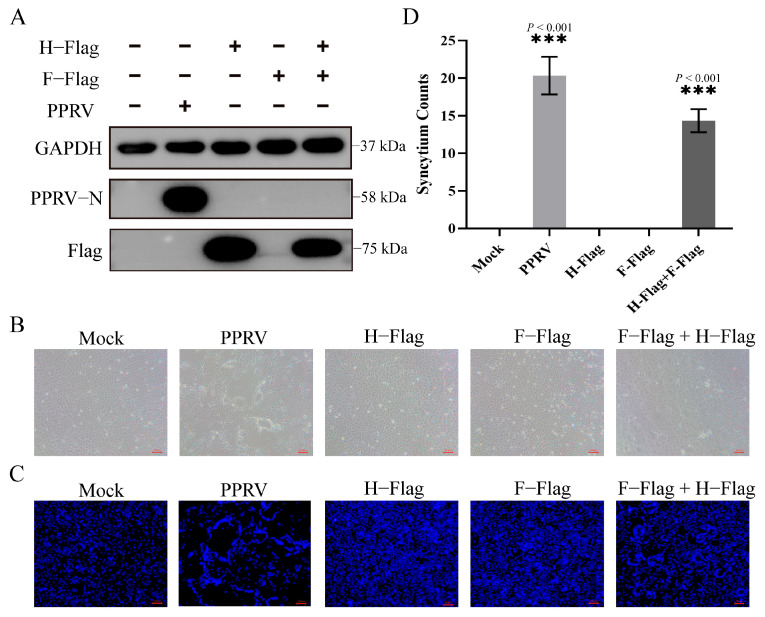
PPRV H and F proteins are required for syncytium formation: (**A**) Western blot analysis confirming the expression of PPRV proteins in each group. The blots were probed with antibodies against PPRV-N, Flag, and GAPDH. (**B**) Representative bright-field microscopy images of syncytium formation in Vero CCL-81-gSLAM cells under different conditions: mock infection, PPRV infection, transfection with H-Flag alone, transfection with F-Flag alone, or co-transfection with F-Flag and H-Flag. Scale bar, 100 μm. (**C**) Immunofluorescence staining of nuclei (DAPI, blue) in Vero CCL-81-gSLAM cells under the same treatments as shown in panel B. Scale bar, 100 μm. (**D**) Quantification of syncytium numbers after the treatments as shown in panel B. Data are representative of three independent experiments and presented as mean ± SD. *** *p* < 0.001.

To identify the viral components essential for PPRV-induced syncytium formation, we examined the roles of H and F proteins, which are known to be critical for viral entry and membrane fusion in paramyxoviruses. We confirmed that both H and F proteins were successfully expressed in cells following plasmid transfection, as verified by Western blotting (Figure 3A). Notably, transfection with H or F alone did not induce appreciable syncytium formation, whereas co-transfection of both genes resulted in robust syncytium formation (Figure 3B–D). These results demonstrate that H and F proteins act cooperatively to promote syncytium formation, indicating their critical roles in PPRV-induced cell-to-cell fusion.

### 3.3. Inhibition of Syncytium Formation Significantly Suppresses PPRV Replication

**Figure 4:**
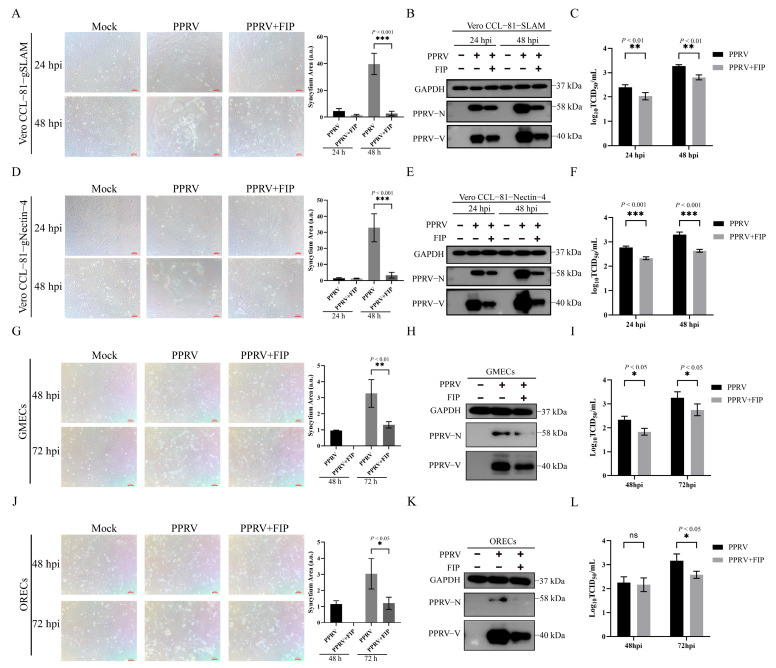
Inhibition of syncytium formation impairs PPRV replication in multiple cell types: (**A**,**D**,**G**,**J**) Representative bright-field microscopic images of syncytium formation in PPRV-infected Vero CCL-81-gSLAM (**A**), Vero CCL-81-gNectin-4 (**D**), GMEC (**G**), and OREC (**J**) cells with or without FIP treatment. (**B**,**E**,**H**,**K**) The effect of FIP treatment on the expression of viral proteins N and V was detected by Western blotting in PPRV-infected Vero CCL-81-gSLAM (**B**), Vero CCL-81-gNectin-4 (**E**), GMEC (**H**), and OREC (**K**) cells. (**C**,**F**,**I**,**L**) The effect of FIP treatment on virus titer was detected by TCID_50_ assay in PPRV-infected Vero CCL-81-gSLAM (**C**), Vero CCL-81-gNectin-4 (**F**), GMEC (**I**), and OREC (**L**) cells. Data are representative of three independent experiments and presented as mean ± SD. *** *p* < 0.001; ** *p* < 0.01; * *p* < 0.05; ns, *p* > 0.05.

Given that PPRV infection induces syncytium formation in various cell types, we further investigated the impact of syncytium formation on viral replication. Fusion inhibitory peptide (FIP), which specifically inhibits the membrane fusion activity of viral glycoproteins, was used to suppress syncytium formation. The results showed that FIP treatment significantly inhibited syncytium formation in Vero CCL-81-gSLAM (Figure 4A) and Vero CCL-81-gNectin-4 (Figure 4D) cells. Importantly, compared with the control group, FIP treatment significantly reduced the expression of PPRV N and V (Figure 4B,E) as well as the viral titers (Figure 4C,F) in the above model cells, indicating that inhibition of syncytium formation markedly attenuates PPRV replication in these cells. Furthermore, we examined the effect of FIP on viral replication in GMECs and ORECs. Similar to the observations in model cells, FIP treatment suppressed syncytium formation in GMECs (Figure 4G) and ORECs (Figure 4J) and also significantly decreased the expression of PPRV N and V (Figure 4H,K) and viral titers (Figure 4I,L). Collectively, these results demonstrate that inhibiting syncytium formation significantly suppresses PPRV replication.

### 3.4. PPRV Infection Induces Syncytium Formation via RhoA-Rock1 Signaling Pathway

**Figure 5:**
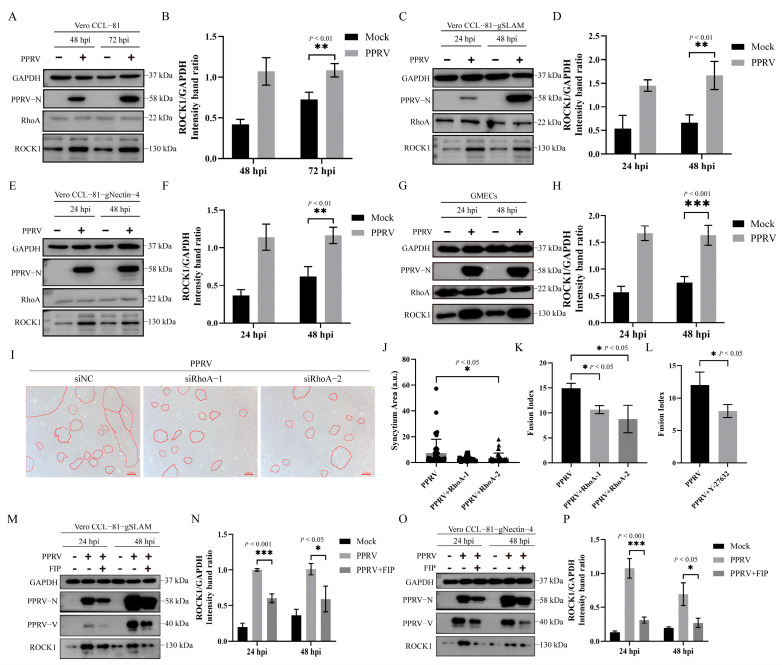
PPRV infection induces syncytium formation via the RhoA-Rock1 signaling pathway: (**A**–**H**) Western blotting analysis of RhoA and Rock1 protein levels in Vero CCL-81 (**A**,**B**), Vero CCL-81-gSLAM (**C**,**D**), Vero CCL-81-gNectin-4 (**E**,**F**), and GMEC (**G**,**H**) cells at the indicated time points upon PPRV infection. (**I**) Representative bright-field microscopic images of syncytium formation in Vero CCL-81-gSLAM cells transfected with siNC or siRNAs targeting RhoA (siRhoA-1 and siRhoA-2) upon PPRV infection. Syncytia are visualized in red lines. (**J**,**K**) Relative syncytium area (**J**) and fusion index (**K**) in panel I. (**L**) The fusion index was measured following PPRV infection with or without Y-27632 treatment. (**M**–**P**) The Rock1 protein levels in Vero CCL-81-gSLAM and Vero CCL-81-gNectin-4 were determined by Western blotting upon PPRV infection with or without Y-27632 treatment. Data are representative of three independent experiments and presented as mean ± SD. *** *p* < 0.001; ** *p* < 0.01; * *p* < 0.05.

The RhoA-Rock1 signaling pathway plays a critical regulatory role in syncytium formation in a variety of viruses; thus, we investigated whether PPRV infection induces syncytium formation by modulating this pathway. As a result, we found that PPRV infection significantly upregulated Rock1 expression, whereas the expression of RhoA showed no significant change in Vero CCL-81 (Figure 5A,B), Vero CCL-81-gSLAM (Figure 5C,D), and Vero CCL-81-gNectin-4 (Figure 5E,F) cells. More importantly, consistent with the observations in model cells, PPRV infection markedly increased Rock1 expression in GMECs (Figure 5G,H), preliminarily indicating that PPRV activates the RhoA-Rock1 signaling pathway across multiple cell types. To further validate the relationship between the RhoA-Rock1 signaling pathway and PPRV-induced syncytium formation, we employed RNA interference (RNAi) to knock down RhoA expression. RhoA knockdown significantly reduced the fusion index and area of syncytia following PPRV infection (Figure 5I–K). Similarly, treatment with Y-27632, a specific inhibitor of Rock1, also diminished PPRV infection-induced syncytium formation (Figure 5L). These results indicate that PPRV promotes syncytium formation by activating the RhoA-Rock1 signaling pathway. In addition, we further examined the effect of FIP treatment on the RhoA-Rock1 signaling pathway. Compared with the control group, FIP treatment significantly reduced Rock1 expression and consequently inhibited syncytium formation (Figure 5M–P), indicating that inhibition of syncytium formation suppressed the activation of the RhoA-Rock1 signaling pathway. Collectively, these findings demonstrate that PPRV infection-induced syncytium formation is strongly associated with the RhoA-Rock1 signaling pathway.

### 3.5. Inhibition of the RhoA-Rock1 Signaling Pathway Significantly Attenuates PPRV Replication

**Figure 6:**
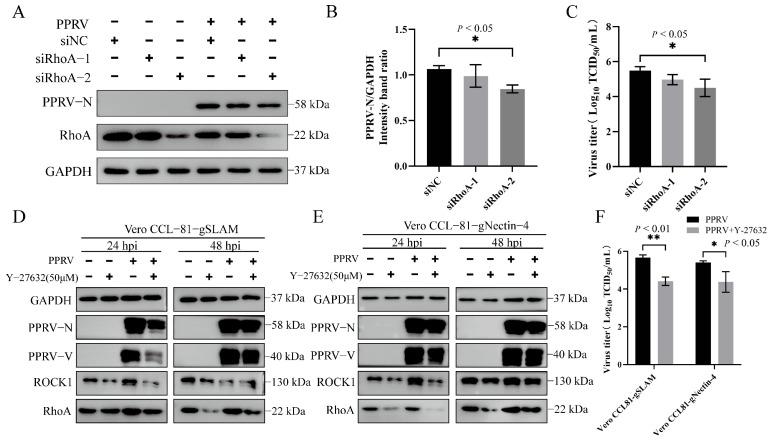
Inhibition of the RhoA-Rock1 pathway suppresses PPRV replication: (**A**) Western blotting analysis of PPRV-N and RhoA protein levels in Vero CCL-81-gSLAM cells transfected with siNC or siRhoA (siRhoA-1 or siRhoA-2) upon PPRV infection. (**B**) The relative expression level of PPRV-N in panel A. (**C**) The effect of RhoA knockdown on virus titer was detected by TCID_50_ assay in PPRV-infected Vero CCL-81-gSLAM cells. (**D**,**E**) Western blotting analysis of PPRV-N, PPRV-V, Rock1, and RhoA protein levels upon PPRV infection in Vero CCL-81-gSLAM (**D**) and Vero CCL-81-gNectin-4 (**E**) with or without Y-27632 treatment. (**F**) The effect of Y-27632 treatment on virus titer was detected by TCID_50_ assay in PPRV-infected Vero CCL-81-gSLAM (**D**) and Vero CCL-81-gNectin-4 (**E**) cells. Data are representative of three independent experiments and presented as mean ± SD. ** *p* < 0.01; * *p* < 0.05.

Based on our finding that PPRV induces syncytium formation via the RhoA-Rock1 signaling pathway, we further investigated the role of this pathway in viral replication. The results indicate that RhoA knockdown significantly reduced the expression of the viral N protein (Figure 6A,B) and viral titers (Figure 6C) in the culture medium. In addition, treatment with Y-27632 reduced the expression levels of both RhoA and Rock1. More importantly, Y-27632 treatment significantly downregulated the expression of the viral N and V proteins (Figure 6D,E) and simultaneously decreased viral titers (Figure 6F). Collectively, these results demonstrate that PPRV infection promotes viral replication by inducing syncytium formation, which is strongly associated with altered Rock1 expression and RhoA-Rock1-dependent modulation.

Taken together, our findings demonstrate that PPRV induces syncytium formation in multiple cell types and identify H and F as key viral determinants. Furthermore, we establish that PPRV-induced syncytium formation is strongly associated with altered Rock1 expression and RhoA-Rock1-dependent modulation and that inhibition of syncytium formation markedly suppresses viral replication.

## 4. Discussion

Syncytium formation, a characteristic CPE associated with a range of viral infections, constitutes a major cause of tissue damage. Numerous studies have confirmed that PPRV infection can induce obvious syncytium formation in caprine primary cells; however, these syncytia are relatively small and limited in number. Moreover, the proliferation of PPRV in caprine primary cells is also relatively weak, which greatly restricts research into the mechanism of syncytium formation induced by PPRV infection. It has been reported that a stable cell line expressing the canine SLAM receptor was constructed for the isolation and cultivation of CDV [16]. Additionally, Vero-SLAM cells expressing human SLAM have been used for screening anti-measles virus drugs [17]. Therefore, to deeply investigate the molecular mechanism of syncytium formation induced by PPRV infection, we selected the PPRV-susceptible model cell line Vero CCL-81 and constructed stable cell lines expressing goat SLAM and Nectin-4 (key receptors for PPRV entry), designated Vero CCL-81-gSLAM and Vero CCL-81-gNectin-4, respectively. After PPRV infection, these cell lines exhibited typical syncytia with increased numbers and larger areas, making them ideal research models for exploring the mechanism of syncytium formation induced by PPRV infection.

Multiple viral infections promote their own replication by inducing syncytium formation. It has been reported that the SARS-CoV-2 D614G variant enhances its replication by inducing stronger syncytium formation [6]. Human respiratory syncytial virus (RSV) and human parainfluenza virus type 3 (PIV-3) promote their replication through syncytium formation [18]. Our previous study found that FIP treatment inhibits syncytium formation in PPRV-infected goat endometrial epithelial cells (EECs), thereby suppressing viral replication [9]. However, syncytium formation in EECs upon PPRV inoculation is relatively limited. Here, we further selected Vero CCL-81-gSLAM and Vero CCL-81-gNectin-4 cells as model cell systems and found that FIP treatment significantly inhibited syncytium formation in these cells and downregulated viral replication levels. More importantly, consistent phenotypes were observed in two other host cells, GMECs and ORECs. Thus, syncytium formation is crucial for PPRV replication. Notably, how syncytia regulate PPRV replication remains to be further investigated.

Given the important role of syncytia in the pathogenesis and replication of various viruses, in-depth elucidation of the molecular mechanisms underlying virus-induced syncytium formation is of great significance for viral disease prevention and control. To date, several key signaling pathways and host factors involved in virus-induced syncytium formation have been identified. It has been reported that the spike (S) protein of Swine Acute Diarrhea Syndrome Coronavirus (SADS-CoV) promotes cellular cholesterol accumulation to induce syncytium formation through the ITGB1-mediated PI3K/AKT/AMPK pathway [19]. RSV and ARV induce syncytium formation by activating the RhoA signaling pathway [12,13,20]. gga-miR-30c-5p inhibits ARV infection-induced autophagy by targeting autophagy-related 5 (ATG5), thereby suppressing virus-induced syncytium formation [21]. In addition, measles virus, which belongs to the same genus as PPRV, relies on the action of CADM1/2 to regulate membrane fusion and promote syncytium formation [22]. Collectively, these findings indicate that syncytium formation is closely associated with cytoskeletal rearrangement and intercellular adhesion and exhibits virus-specific characteristics. Here, we discovered that PPRV induces syncytium formation and promotes its own replication by activating the RhoA-Rock1 signaling pathway. Interestingly, RhoA expression did not change significantly upon PPRV infection, but knockdown of RhoA significantly reduced both the number and size of syncytia. This may be due to the binding of the PPRV fusion glycoproteins H and F to RhoA, which regulates subsequent syncytium formation without affecting RhoA expression. Moreover, although inhibiting the RhoA-Rock1 signaling pathway decreases both the number and size of syncytia induced by PPRV infection, it fails to fully block syncytium formation, indicating that other unknown host factors contribute to this process.

The formation of syncytia induced by viral infection primarily relies on the binding of viral fusion proteins to host cell membrane proteins, thereby promoting cell membrane fusion through the rearrangement of host cytoskeletal proteins. Current studies have shown that the capsid surface proteins of various viruses are key viral components that induce syncytium formation, for example, the spike (S) protein of SARS-CoV-2 [23], the gB protein of Herpes simplex virus (HSV) [24], the F protein of Nipah virus (NiV) [25], and the F protein of RSV [26]. Here, we identified that during PPRV infection, the viral surface fusion proteins H and F are the key viral proteins that induce syncytium formation. More importantly, neither H nor F alone can induce syncytium formation; instead, they rely on the cooperative action of both. We hypothesize that the H protein primarily binds to specific receptors on the host cell surface (such as SLAM or nectin-4), thereby anchoring the virus to the target cell and creating the necessary spatial and physical conditions for subsequent F protein-mediated membrane fusion, which serves as an upstream event that activates the RhoA-Rock1 signaling pathway. Specifically, the interaction between the H protein and its receptors creates the necessary conditions for viral entry, whereas the F protein-mediated fusion process serves as a core switch that activates the key signaling molecule RhoA, for example, by binding RhoA to activate its enzymatic activity. Furthermore, the H-induced binding of the viral particle to the host cell membrane may trigger the cleavage or conformational change of F, thereby initiating the F protein-mediated membrane fusion process; however, the specific molecular mechanism requires further verification. In addition, syncytium formation induced by viral infection is closely associated with the oligomerization and glycosylation modifications of membrane fusion proteins. It has been reported that in the case of canine distemper virus (CDV), another morbillivirus, H forms a typical tetrameric structure during infection [27]. Syncytium formation induced by RSV infection depends on the N-glycosylation modification of the viral fusion protein [28]. However, whether PPRV forms a similar structure and its precise spatial conformation during the induction of membrane fusion requires further investigation.

## 5. Conclusions

In summary, our findings demonstrate that PPRV infection induces syncytium formation and promotes viral replication by activating the RhoA-Rock1 signaling pathway, with the H and F proteins serving as the key viral proteins responsible for syncytium formation. These results will further elucidate the pathogenic mechanism of PPRV and provide an important theoretical basis for the development of novel antiviral strategies.

## Data Availability

All data generated or analyzed during this study are included in this published article. Additional information is available upon request from the corresponding authors.
